# Critical length scale controls adhesive wear mechanisms

**DOI:** 10.1038/ncomms11816

**Published:** 2016-06-06

**Authors:** Ramin Aghababaei, Derek H. Warner, Jean-Francois Molinari

**Affiliations:** 1 Institute of Civil Engineering, Institute of Materials Science and Engineering, Ecole Polytechnique Fédérale de Lausanne (EPFL), CH 1015 Lausanne, Switzerland; 2 School of Civil and Environmental Engineering, Cornell University, 373 Hollister Hall, Ithaca, New York 14853, USA

## Abstract

The adhesive wear process remains one of the least understood areas of mechanics. While it has long been established that adhesive wear is a direct result of contacting surface asperities, an agreed upon understanding of how contacting asperities lead to wear debris particle has remained elusive. This has restricted adhesive wear prediction to empirical models with limited transferability. Here we show that discrepant observations and predictions of two distinct adhesive wear mechanisms can be reconciled into a unified framework. Using atomistic simulations with model interatomic potentials, we reveal a transition in the asperity wear mechanism when contact junctions fall below a critical length scale. A simple analytic model is formulated to predict the transition in both the simulation results and experiments. This new understanding may help expand use of computer modelling to explore adhesive wear processes and to advance physics-based wear laws without empirical coefficients.

## Abstract (web summary)

Adhesive wear can proceed through qualitatively different mechanisms, with conflicting results in the literature. Here the authors observe a transition between two regimes in simulations using model interatomic potentials, allowing development of a simple analytical theory to describe past results.

## Graphical abstract

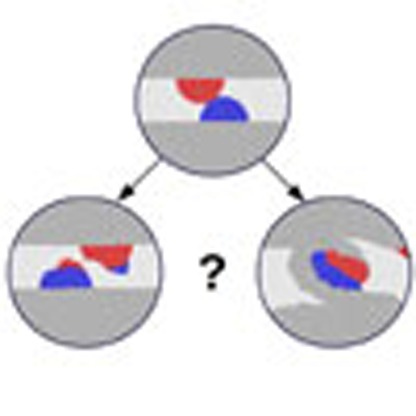

Adhesive wear commonly occurs at the sliding contact between materials with comparable hardness in the presence of a strong adhesive force^1^,^2^. During sliding, welding actions occur between a limited number of surface asperities which undergo large plastic deformation. In 1946, Holm^3^ proposed one of the first models to picture the adhesive wear mechanism and material removal at the asperity level. The model assumed that those plastically deformed surface asperities are worn away gradually by the removal of atoms as the two surfaces slide. Thanks to technological progress, this mechanism has since been observed in atomic force microscopy (AFM) experiments^4^,^5^,^6^,^7^,^8^,^9^ and predicted by atomistic simulations^10^,^11^,^12^. Despite these confirming results, the role of the atom removal mechanism in typical adhesive wear is not widely agreed upon^13^. Alternatively, Archard proposed that adhesive wear occurs by fracture and the creation of debris particles^14^, an idea that is extensively confirmed by experimental observations at different scales^15^,^16^,^17^,^18^. In support of Archard's model, the friction and wear response of many tribological systems reaches a steady state, a feature that can be easily understood with Archard's wear mechanism, but not with Holm's gradual smoothing mechanism that implies the flattening of surfaces and eventually cold welding. Ultimately, Archard and Holm's wear models are empirical and the controlling wear mechanisms are not clear.

One reason that a widely agreed upon understanding of the adhesive wear mechanism has not been reached, is that direct modelling of the adhesive wear process presents a substantial challenge. Continuum models are limited in that they cannot explicitly simulate the wear process due to the complexity of the severe deformation, fracture and contact associated with wear debris^19^. Alternatively, atomistic modelling can handle these features, but is often disconnected from reality due to a disparity of scale and the challenge of accurately representing the interatomic interactions of real material systems. Previous atomistic modelling studies of the adhesive wear predict a continual smoothing of surfaces rather than a steady-state wear regime with debris particles^10^,^11^,^12^,^20^,^21^,^22^,^23^; inconsistent with common experimental observations. This discrepancy is schematically depicted in Fig. 1.

Here we present atomistic simulations that capture, to our knowledge, for the first time the fracture-induced debris formation during the adhesive collision between surface asperities. This is achieved by developing simple two-dimensional (2D) model potentials with tuneable inelastic properties which can then be linked to the macroscopic behaviour of real materials, where details of the potentials no longer appear. A systematic set of atomistic simulations with these potentials reveals a characteristic length scale that controls the adhesive wear mechanisms at the asperity level. This length scale provides a critical adhesive junction size, where bigger junctions produce wear debris by fracture while smaller ones smooth out plastically. On the basis of this observation, we formulate a simple analytical model that predicts the transition in the asperity-level adhesive wear mechanisms in both the simulation results and experiments.

## Results

### Development of model potentials

We first consider that the continual smoothing trend predicted by previous atomistic models is realistic, as the simulations involve pure materials in vacuum with at most nanometre surface roughness. Then, we hypothesize that changing the material properties in an atomistic simulation could lead to wear debris formation and ultimately a steady-state sliding regime, that is, sustained debris formation. Specifically, we hypothesize that increasing hardness should favour the formation of sustained debris particles in a tribological system, with all other properties remaining constant. This idea is consistent with the pervasive theme in the tribology literature that the ratio of surface energy over hardness plays an important role in determining tribological response^24^,^25^,^26^,^27^,^28^,^29^. Motivated by this argument, we design a family of model interatomic potentials to modify this ratio, while keeping other properties such as lattice and elastic constants unchanged.

**Figure 2:**
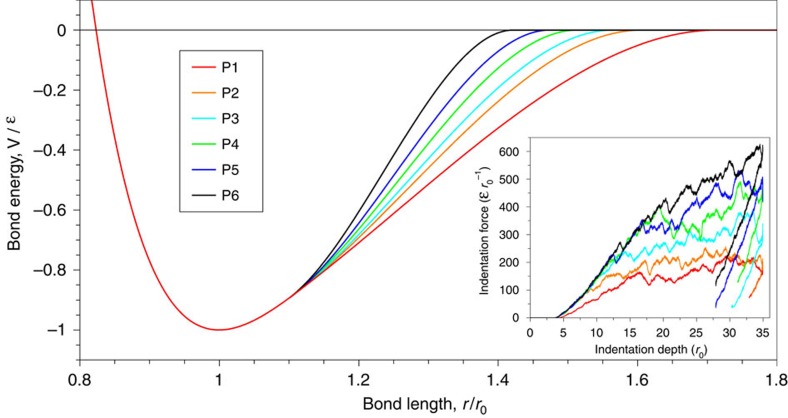
Model interatomic potentials and the corresponding indentation responses. The bond energy versus atomic bond length is plotted for different cutoff radius (see also equation (4)). Bond energy and length are normalized by the depth of the potential well (*ɛ*) and the equilibrium bond length (*r*_0_), respectively. The hardness/brittleness of the potentials increases from P1 to P6. The inset shows the indentation responses of the potentials, demonstrating the differing hardness and constant elastic modulus (upon unloading). See Supplementary Fig. 2 for a quantitative analysis of the potentials hardness.

Within the domain of an atomistic simulation, hardness is controlled by dislocation nucleation, which is governed by the unstable stacking fault energy, *γ*_usf_ (ref. 30). The unstable stacking fault energy is determined by the energy of highly stretched bonds in relation to the tail of the interatomic potential. Focusing on a simple atomistic model with only nearest-neighbour pairwise interactions, the tail of the interatomic potential can be modified without influencing the surface energy (*γ*_surf_), elastic parameters and lattice constant. Here we modified the long-range character of the Morse potential^31^ without disturbing the short-range interactions (elastic properties), as described in ‘Methods section'. Figure 2 shows the model potentials (named P1–P6) and the corresponding indentation responses, that confirms the differing hardness and constant elastic modulus (see Supplementary Figs 1 and 2). A detailed quantitative analysis of the model potentials are provided in Supplementary Table 1. A second set of potentials is developed to mimic the interfacial adhesion between the two sliding surfaces. These potentials are denoted as P6-1 through P6-6 and differ by a simple scaling of the bond energy (see Supplementary Fig. 3 and Supplementary Table 2). When not explicitly stated, the same potential used within the bulk (P1–P6) is used between surfaces, giving the junction between the two surfaces the same strength as the bulk.

### Asperity-level simulations of adhesive wear

We perform a large number of atomistic simulations with different geometrical configurations (that is, interlocking asperities and single-asperity sliding on a flat surface), boundary conditions, and bulk and surface properties (see Supplementary Fig. 4). Further details of the simulations are provided in the ‘Methods section'. It is important to emphasize that the simulation setup utilized here simplifies many of the complexities of real tribological systems, with the goal of providing scientific insight into the asperity-level wear mechanism. This work is focused on the adhesive wear regime, noting that the mechanisms in other wear regimes may be distinctly different, for example, abrasive^32^,^33^,^34^. In abrasive wear, a much harder material/body removes material from a softer surface through a plowing or gouging process, as opposed to a sticking and tearing mechanism between two bodies of comparable hardness in adhesive wear. Complexities such as oxidation, tribomaterial layers, and lubrication are not explicitly included, but would be partially included in our modelling framework through their influence on the surface energy, junction and bulk strength^35^,^36^,^37^.

**Figure 3:**
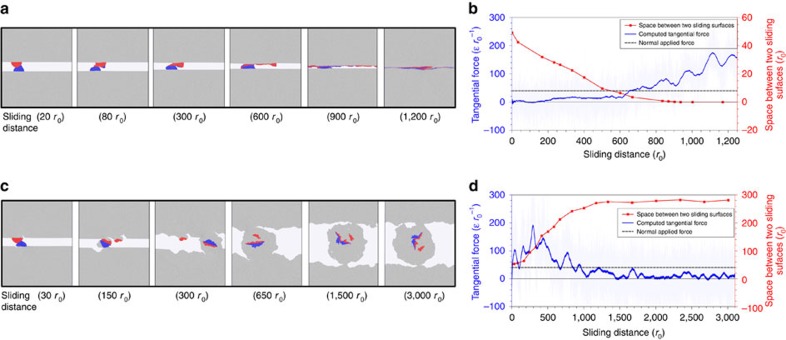
Simulated colliding asperities reveal two distinct wear mechanisms. (**a**) Snapshots of simulation with the most ductile potential (P1) revealing the continual asperity smoothing mechanism that eventually leads to cold welding are shown. (**b**) Depiction of the corresponding evolution of frictional force and spacing between sliding surfaces confirming an increase in contact area. (**c**) Snapshots of simulation with the most brittle potential (P6) revealing the debris formation wear mechanism are shown. The corresponding evolution of frictional force and spacing between sliding surfaces are shown in **d**, demonstrating a steady-state regime with respect to the particle size, surface roughness and frictional force. Both tangential force and sliding distance are given in reduced Lennard-Jones units. The colouring of atoms is artificial and for better visualization of the wear process.

Inspired by our atomistic simulations, here, we elaborate two experimentally observed pictures of adhesive wear mechanisms, that is, gradual plastic smoothing and fracture-induced debris particle. Figure 3a shows images from a dry sliding simulation with the most ductile potential with full adhesion (P1). As the two pieces slide, a strong adhesive bond is established between the contacting asperities, leading to severe plastic shearing at the junction, as described by the classical Bowden and Tabor model^24^ and observed in recent AFM experiments^6^,^38^. Upon sliding, a continual smoothing of the asperities, consistent with Holm's model^3^, occurs with atoms exchanging between the surfaces, as observed in nanoscale experiments^4^,^6^,^38^ and previous molecular dynamics simulations^10^,^11^,^20^. Eventually, the surfaces become flattened to the extent that cold welding occurs along the entire surface. Figure 3b corroborates this story, showing an increasing tangential force with sliding distance, due to an increasing contact area. Further, the gradual reduction of the vertical distance between the sliding pieces is consistent with the continual smoothing of the asperities.

The outcome of a dry sliding simulation with the most brittle potential and full adhesion (P6) is shown in Fig. 3c. During sliding, a strong adhesive bond is also established between the contacting asperities, leading to a build-up and subsequent release of stored elastic energy (corresponding to the first peak in the tangential force curve in Fig. 3d). The release of stored elastic energy is due to the formation of cracks at the bases of the asperities. As sliding continues, the cracks propagate and eventually reach the surfaces which result in a wear debris particle. This observation is consistent with the general picture of debris formation in adhesive wear^1^,^16^. With further sliding, the debris particle rolls and increases in size. Ultimately, the simulation reaches a steady state with respect to the particle size, surface roughness and frictional force (Fig. 3d). Together, the formation of debris and the subsequent steady-state rolling behaviour provide a mechanism capable of explaining the macroscopic phenomena of steady-state friction and wear. To the best of our knowledge, this is the first time that this mechanism has been directly observed in a simulation. The observation is consistent with Archard's model^14^ and the formation of cylindrical rolls of wear debris in experiments on ceramics and rocks^39^,^40^,^41^,^42^. In addition, the fracture-induced debris formation mechanism and the corresponding evolution of the tangential force (Fig. 3d) are coherent with previous analytical and experimental observations^1^,^15^,^16^.

Examining a range of simulations parameters, we found that the asperity deformation mechanism was not significantly affected by sliding velocity, initial asperity shape (examining rectangular through half sine shapes) and asperity configuration (examining systems with a single or interlocking asperities), (see ‘Methods section'). In contrast, the size of the asperity contact junction, and the strength of the adhesive bond between the pieces (studied using potentials P6-1 through P6-6) were found to control the debris formation in adhesive wear. Supplementary Fig. 5 presents individual contributions of bulk and interfacial shear strength. Ultimately, hard/brittle materials with large asperities and strong adhesive bonding at the asperity contact junctions favoured the debris formation mechanism over the asperity smoothing mechanism, confirming the hypothesis stated in the introduction.

### Analytical model

Inspired by previous theoretical insights^26^,^28^,^43^ and experimental observations^4^,^6^,^18^,^44^, we find that this result can be predicted with an analytic model based on the minimization of net configuration energy. Specifically, the model assumes that a stable wear debris particle will form when its configuration becomes energetically favoured over that of a strained junction, that is, ample energy is present at the sliding surfaces to quickly overcome any kinetic barriers. This assumption is consistent with pre-existing ideas in the literature^27^,^45^ and the simulation results, in that the occurrence of debris formation and sustained rolling is largely independent of the details of the starting configuration.

The model considers the change in energy associated with the formation of a debris particle relative to an asperity junction loaded to its elastic limit in shear. Considering a three-dimensional (3D) general case, the elastic energy released by the creation of a debris particle can be written as


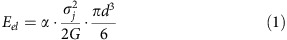


where *G* is the shear modulus and *σ*_*j*_ is the shear strength of the junction, a value assumed to be the lesser of the bulk material shear strength and the adhesive junction shear strength. A spherical particle of the same diameter, *d*, as the junction size is considered, where the factor *α* accounts for the particle shape and stress distribution. The stress distribution near the junction is assumed to be relatively uniform due to the large amounts of plastic deformation. A detailed analysis of our atomistic simulations confirms the scalability of the released elastic energy with junction size (see Supplementary Fig. 6), which is consistent with the literature^46^,^47^,^48^.

**Figure 4:**
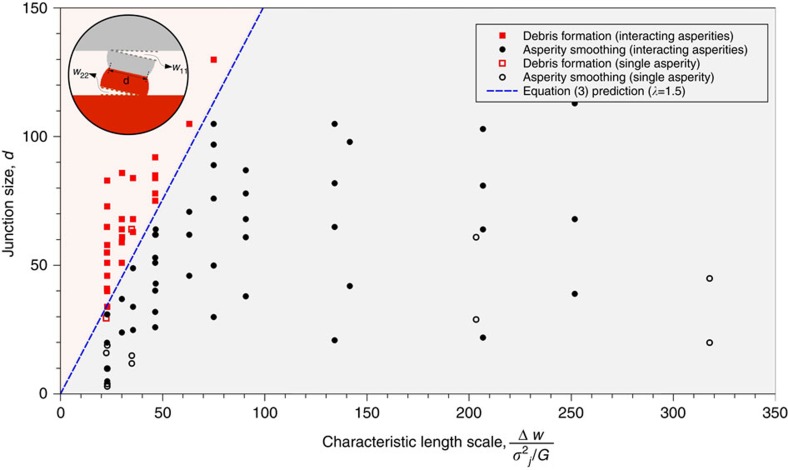
Adhesive wear mechanisms map. Summary of all atomistic simulations result with different asperity size, shape, material hardness, interfacial adhesion, applied load, sliding velocity and boundary conditions (see also ‘Methods section'). These results show a sharp transition in wear mechanism at the characteristic length scale (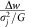) predicted by equation (3). Both junction size and characteristic length scale are given in a reduced Lennard-Jones length unit. See Supplementary Fig. 9 for detailed information of atomistic results.

The adhesive energy to debond the two asperities from the sliding surfaces and create new free surfaces in both solids can be written as


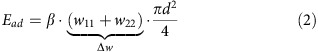


where *w*_11_ and *w*_22_ are the energy associates to newly created free surfaces because of a unit area of crack growth in each sliding body. The factor *β* is the ratio of the debonded area underneath each asperity to the junction area (see the inset of Fig. 4). Here, we assumed that both surfaces equally contribute to the formation of debris, while in case of sliding between non-identical materials, a bigger contribution from the softer material is expected.

The existence of sustained debris particles is then predicted when *E*_*el*_>=*E*_*ad*_. This subsequently entails the existence of a critical length scale that governs the adhesive wear mechanism


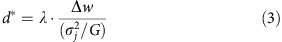


whereby debris particles will form when *d*>d*, with *λ* being a shape factor combining contributions of all geometrical factors. Assuming *α*=*β*=1, we obtain *λ*=8/*π* and *λ*=3, corresponding to the removal of an idealized 2D circular and 3D spherical debris respectively. While the presented model is constructed based on the minimization of net configuration energy, a similar criterion may be considered based on a crack initiation model^30^,^49^, in which a detailed kinetics of crack growth and other dissipative mechanisms (for example, plasticity) could be taken into account.

### Transition in adhesive wear mechanisms

Predictions of the proposed model are plotted in the form of a wear mechanism map in Fig. 4. Superimposed on the map, are the results from a large set of atomistic simulations examining different initial asperity sizes, shapes (that is, semicircular and partial circular segment, triangular, rectangular and half sine, Supplementary Fig. 7) and configurations (that is, single versus interlocking asperities, Supplementary Fig. 8), system dimensions, applied loads, sliding velocities, boundary conditions and various body and interfacial potentials (see Supplementary Fig. 9). The figure shows that the atomistic simulation results are remarkably well explained by the proposed model specialized to 2D with *λ*=1.5, predicting the transition in mechanisms as a function of junction strength, work of adhesion and junction size. For example, both the simulation results and the model predict the continual smoothing mechanism when the adhesion between contacting materials goes to zero.

In the simulations, the junction size is measured when the tangential force first peaks. The elastic modulus and junction strength are obtained by a separate set of molecular dynamics simulations (see Supplementary Table 1). The adhesion energy per unit are of crack is estimated from the surface-free energy (that is, Δ*w*=*w*_11_+*w*_22_=4*γ*_*surf*_). Note that *λ*=1.5 is close to the idealized case of complete removal of 2D circular asperities, which yields *λ*=8/*π* (Fig. 4 insert).

**Figure 5:**
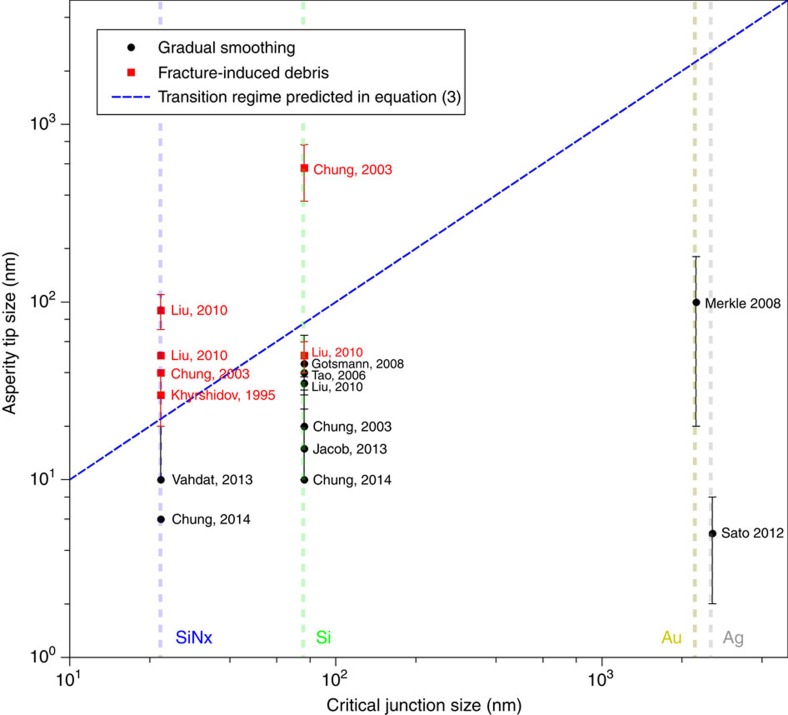
Critical size prediction versus results from AFM wear experiments. A compilation of recent adhesive AFM wear experiments where two adhesive wear mechanisms have been observed, that is, gradual smoothing^4^,^5^,^6^,^7^,^9^,^18^,^50^,^51^ and fracture-induced debris formation^17^,^18^,^52^. Detailed information of AFM wear experiments are provided in Supplementary Table 5. The *x* axis represents the critical junction size, estimated by equation (3) (see Supplementary Table 4). The *y* axis shows an estimation of the real junction size (AFM tip diameter). The blue dash line shows the transition regime, predicted by equation (3) and in agreement with the experimental observations. See Supplementary Tables 4 and 5 for further information.

The analytic model can also be directly compared with adhesive AFM wear experiments, where the AFM tip has been reported to wear due to adhesive forces via both gradual smoothing^4^,^5^,^6^,^7^,^9^,^18^,^50^,^51^ and the creation of fracture-induced debris^17^,^18^,^52^ similar to our single-asperity simulations (see Supplementary Figs 8 and 10). Inserting values for the junction size, strength and shear modulus into the analytic model (as detailed in the Supplementary Tables 3 and 5), we find that the model accurately predicts the operative mechanism in almost every case (Fig. 5). The ability of the model to predict the experimentally observed behaviour, not only validates its utility but provides an explanation for the discrepant AFM wear experiment observations.

## Discussion

The proposed model predicts the sustained wear debris formation at junctions with sizes above hundreds of nanometres and tens of microns in ceramics and metals, respectively (see Supplementary Table 4). This finding explains why sustained wear debris has not been observed in previous atomistic simulations of adhesive wear^10^,^11^,^12^,^20^,^21^,^22^,^23^, where the junction is too small/weak to form sustained roller debris by fracture. This also highlights a need for robust multi-scale simulation frameworks for modelling adhesive wear. The model predictions explicate the utility of the simple interatomic potentials introduced here, which allowed debris formation to be studied with atomistic simulation. We have confirmed this conclusion by performing multimillion-atom 3D simulations with several well-established interatomic potentials (see ‘Methods section' and Supplementary Fig. 11). As predicted by the proposed model, stable wear debris is not formed in these simulations because of their length scale and material properties, a result consistent with the most recent 3D adhesive wear simulations using a state-of-the-art interatomic potential^23^. We emphasize that, while this calculation predicts fracture-induced debris formation for junction sizes above tens of microns in metals, consistent with experimental observations^16^, other wear mechanisms such as surface folding and delamination^34^ may occur at lower scales due to subsurface dislocation structure and/or surface oxidation.

Beyond single-asperity interactions, the presented model also illuminates several key macroscopically observable features of wear. It predicts a wear coefficient (the probability of debris formation from asperity contacts^14^) of <1, considering that a distribution of junction sizes will exist^53^ and only a fraction of those junctions will be of sufficient size to create wear debris. Second, building upon this point, the model predicts that an increase in d* will significantly decrease the rate of wear debris formation. Thus, the model yields a superlinear decrease in wear particle formation rate with a decrease in the friction coefficient, *μ*, consistent with experience, considering that the junction strength, *σ*_*j*_, can be estimated as *μσ*_*y*_ (ref. 24), where *σ*_*y*_ is the yield stress.

In summary, we propose a set of model potentials which allows the simulation of two possible adhesive wear mechanisms at the asperity level, by tuning the critical junction size. This finding provides a unified mechanistic basis for Holm and Archard's adhesive wear models. We have found that the transition in adhesive wear mechanisms is controlled by three features: the size of the asperity contact junction; the work of adhesion of the bulk material; and the maximum elastic strain energy that can be stored at a contact. An analytical model is introduced based on these simulation results which consistently explains within a single framework discrepant AFM experimental observations that report either fracture-induced debris formation or a continual asperity smoothing mechanism. Our approach demonstrates a route for directly studying the wear process with computer simulation by adjusting the critical length scale in an atomistic simulation. These results have potential relevance to applications that involve sliding contact, from traditional engineering machine wear to bioimplants, NEMS/MEMS devices and earthquakes.

## Methods

### Interatomic potentials and corresponding physical properties

At the atomic scale, the competition between the brittle/ductile response is controlled by the surface and unstable stacking fault energy, *γ*_surf_ and *γ*_usf_, respectively. For a nearest-neighbour pairwise potential, *γ*_usf_ can be modified without changing *γ*_surf_, the elastic constants or the lattice constant by changing the tail of the interatomic potential. Therefore, we modified the long-range character of the Morse potential^31^ without disturbing the short-range interactions (elastic properties) as follows


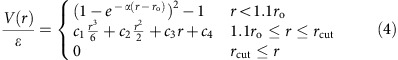


where the *r*_cut_ parameter defines the potential cutoff radius and controls the interaction length scale and *c*_1_–*c*_4_ are parameters to ensure the continuity of bond energy and force. *α* controls the width of the potential, which equal to 3.5 for all the potentials. *r*_o_ is the equilibrium bond distance and *ɛ* is the depth of the potential well. The 1.1 factor ensures constant elastic properties up to 10% strain. This allows us to study the influence of inelastic properties independently of elastic properties.

Supplementary Fig. 1 provides the energy landscape of body potentials for a relative horizontal displacement of two rigid atomic layers parallel to the stacking layer. The peak in each curve represents the corresponding unstable stacking fault energy. To control the interfacial adhesion between the sliding surfaces in our simulations, we also created a set of potentials with bond energy differing by a scalar (Supplementary Fig. 3). Key parameters for both of these sets of potentials are given in Supplementary Tables 1 and 2. For simulations with a lower interfacial adhesion, the junction shear strength, *σ*_*j*_, is estimated from the bulk shear strength multiplied by the ratio 
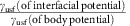
. The accuracy of this approximation has been validated by a separate set of shearing simulations.

To obtain the shear strength associated with each body potential, we performed 2D indentation simulations. To model the non-adhesive contact, we only considered the pure repulsive contribution of the interfacial potential^54^. The indentation response of all potentials are shown in the inset of Fig. 2. To extract the hardness value from these curves, we plot the contact pressure, as the ratio of the indentation force (*P*) to the projected contact area (*A*), versus indentation depth in Fig. 2 (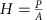) (ref. 55). Surface atoms within the cutoff radius of the interfacial potential are considered to define the atomic area of contact^56^. Accordingly, the projected contact area at the atomic scale is computed using a similar framework as in Ziegenhain *et al*.^57^. As shown in Fig. 2, the peak value in each curve corresponds to the first dislocation nucleation event. Upon continued loading, the stress remains relatively constant as dislocations continue to nucleate. This constant value is taken as the hardness (see Supplementary Table 1). The critical shear strength *τ* corresponding to each potential is estimated as 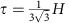 (ref. 58). In simulations with full interfacial adhesion, the junction strength, *σ*_*j*_ is taken equal to *τ*.

According to the well-known Oliver-Pharr method^55^,^59^, the Young's modulus (*E*) can be obtained as 
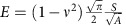
, where *v* is Poisson ratio (∼0.34) and *A* and *S* are the projected area and the slope at the unset of unloading curves (see inset of Fig. 2). This equation gives a Young's modulus of 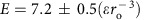 and a shear modulus of 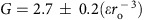 for the body potentials. We confirmed these values by simple tensile and shear simulations.

### Simulation geometry, loading and boundary conditions

All simulations were performed in 2D using LAMMPS (ref. 60). A periodic boundary condition and a sliding velocity were applied in the *x* direction for all the simulations. In the *y* direction, both constant pressure and fixed boundary conditions were examined (Supplementary Fig. 4). Temperature was enforced along two thin layers just outside of this region using a Langevin thermostat with a damping parameter 0.05 in reduced time unit. A Verlet algorithm with a time step 0.0025 (in reduced time unit) is used for numerical integration. All atomistic simulations are visualized by OVITO software^61^. We performed a large set of simulations, which confirmed that the governing wear mechanism at the onset of the asperity contact (that is, gradual plastic smoothing or fracture-induced debris formation) is directly governed by the junction size (Fig. 4), and it is remarkably independent of the simulation box size (Supplementary Table 3), boundary conditions (that is, fixed displacement versus fixed applied pressure, Supplementary Fig. 4), initial asperity shapes (that is, semicircular and partial circular segment, triangular, rectangular and half sine, Supplementary Fig. 7) and geometrical configurations (that is, single-asperity (Supplementary Fig. 8) versus interlocking asperities (Fig. 3)). Note that the final steady-state size of the debris particle depends on the force carried by the junction, which may be influenced by the geometrical factors and boundary conditions (that is, a large force produces a larger particle, consistent with the general picture of adhesive wear).

### Insights from 3D simulations with standard potentials

A wide range of 3D simulations were performed to verify our assertion that debris formation will not occur in 3D simulations with more realistic potentials due to the length scale constraint. The simulations were analogous to the 2D ones described in this manuscript. Specifically, the interactions of two semicircular asperities was examined in a periodic simulation cell under a normal load of 0 and 100 nN and a sliding velocity of 10 m s^−1^. We examined fcc aluminium using the Mendelev *et al*.^62^ EAM potential, bcc iron using the Mendelev *et al*.^63^ EAM potential, and diamond cubic Si using the classical Tersoff/Stillinger-Webber Si potential^64^,^65^. We performed a set of multimillion atom simulations (5–300 million atoms) with asperity diameters between 10 and 45 nm. In no cases was debris formation observed, confirming the prediction of our analytic model (see Supplementary Fig. 11). These simulations and associated complexities further reflect the potential application of the developed 2D potentials. We also emphasize that we are not aware of any interatomic potentials that can accurately capture both the fracture and plastic slip responses of real brittle materials, where the debris formation length scale is expected to be smaller. Details regarding the challenges associated with interatomic potential development for these materials can be found in these works^66^,^67^,^68^ and references therein.

### Compilation of experimental data

The critical junction size for silicon, silicon nitride, silver and gold are computed using equation (3) with the material properties given in Supplementary Table 4. *σ*_*j*_ is estimated from hardness using the relation on page S3. The approach is supported by experiments that indicate a strong adhesive bond between the AFM tip and the substrate^17^,^18^. Note that we only take into account adhesive AFM wear experiments, in which the sliding AFM tip and substrate have comparable hardness (which prevent the penetration of the AFM tip to the substrate) and wear occurs mainly due to adhesive forces (see Supplementary Table 5). While not critical to our conclusions, we choose *λ*=3 corresponding to the removal of an idealized 3D spherical wear particle from an AFM tip (see Supplementary Fig. 10). The average critical junction size, presented on the *x* axis of Fig. 5, is also provided as a bold number in the last column of Supplementary Table 4.

An upper-bound assumption is made on the junction size in the experiments, taking it to be the diameter of the AFM tip. We assume gradual asperity smoothing can transform multi-asperity contact at the AFM tip to single-asperity contact leading to a junction size of approximately the tip diameter (denoted as *d* in Supplementary Fig. 10). This is consistent with our model since the size of the initial atomistic junctions are much smaller than the critical junction size. We also validated this assumption by performing additional single-asperity simulations, in which a random initial atomistic roughness was introduced at the asperity tip. Thus, all points below the blue line in Fig. 5 are predicted to undergo gradual smoothing regardless of the topography of the tip. Considering the agreement between our model and the experimental AFM data, we believe that our upper-bound assumption is reasonable. This new understanding may help expand use of computer modelling to explore adhesive wear processes and to advance physics-based wear laws without empirical coefficients.

### Data availability

The analytical formulation for the developed potentials that used in this study is provided in equation (4) (see Supplementary Tables 1 and 2 for used parameters). Details of the simulations are available within the article and the Supplementary Information. All other data supporting the findings of this study are available from the authors upon request.

## Additional information

**How to cite this article:** Aghababaei, R. *et al*. Critical length scale controls adhesive wear mechanisms. *Nat. Commun.* 7:11816 doi: 10.1038/ncomms11816 (2016).

## Supplementary Material

Supplementary Information Supplementary Figures 1 - 11, Supplementary Tables 1 - 5 and Supplementary References

## Figures and Tables

**Figure 1:**
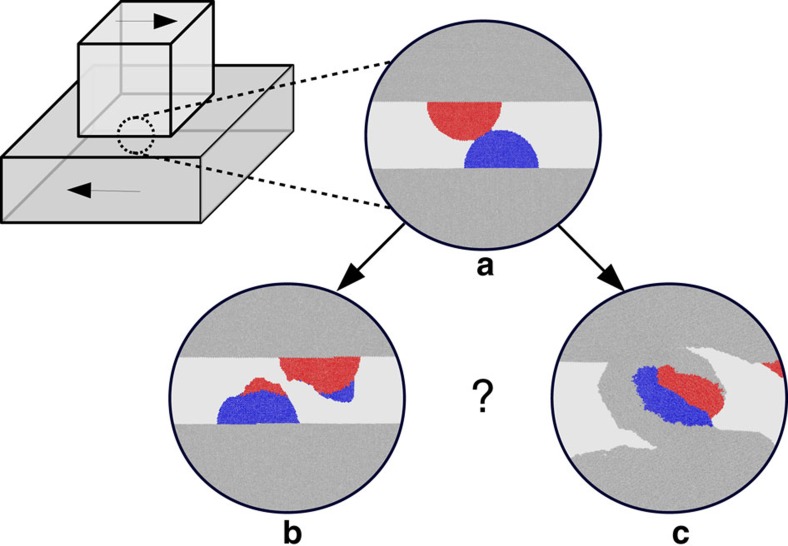
Schematic representation of two possible asperity-level adhesive wear mechanisms. After an adhesive interaction between surface asperities (**a**), the wear process occurs via either (**b**) a gradual smoothing mechanism by plastic deformation^3^ or (**c**) a fracture-induced debris formation mechanism^69^. Both mechanisms have been recently observed in AFM wear experiments^6^,^17^,^18^,^38^. The colouring of atoms is artificial and for better visualization of the wear mechanisms.

## References

[b1] Kato K. & Adachi K. in Modern Tribology Handbook ed. Bhushan B. 273–299CRC Press (2000).

[b2] Burwell J. T. Survey of possible wear mechanisms. Wear 1, 119–141 (1957).

[b3] Holm R. in Electrical Contacts ed. Holm R. Springer 232–242 (1976).

[b4] Merkle A. P. & Marks L. D. Liquid-like tribology of gold studied by *in situ* TEM. Wear 265, 1864–1869 (2008).

[b5] Vahdat V., Grierson D. S., Turner K. T. & Carpick R. W. Mechanics of interaction and atomic-scale wear of amplitude modulation atomic force microscopy probes. ACS Nano 7, 3221–3235 (2013). 23506316 10.1021/nn305901n

[b6] Sato T., Ishida T., Jalabert L. & Fujita H. Real-time transmission electron microscope observation of nanofriction at a single Ag asperity. Nanotechnology 23, 505701 (2012). 23164958 10.1088/0957-4484/23/50/505701

[b7] Jacobs T. D. B. & Carpick R. W. Nanoscale wear as a stress-assisted chemical reaction. Nat. Nanotechnol. 8, 108–112 (2013). 23353678 10.1038/nnano.2012.255

[b8] Bhaskaran H. . Ultralow nanoscale wear through atom-by-atom attrition in silicon-containing diamond-like carbon. Nat. Nanotechnol. 5, 181–185 (2010). 20118919 10.1038/nnano.2010.3

[b9] Gotsmann B. & Lantz M. A. Atomistic wear in a single asperity sliding contact. Phys. Rev. Lett. 101, 125501 (2008). 18851384 10.1103/PhysRevLett.101.125501

[b10] Sorensen M. R., Jacobsen K. W. & Stoltze P. Simulations of atomic-scale sliding friction. Phys. Rev. B 53, 2101–2113 (1996). 10.1103/physrevb.53.2101 9983674

[b11] Zhong J., Shakiba R. & Adams J. B. Molecular dynamics simulation of severe adhesive wear on a rough aluminum substrate. J. Phys. D Appl. Phys 46, 055307 (2013).

[b12] Stoyanov P. . Nanoscale sliding friction phenomena at the interface of diamond-like carbon and tungsten. Acta Mater. 67, 395–408 (2014).

[b13] Schirmeisen A. Wear: one atom after the other. Nat. Nanotechnol. 8, 81–82 (2013). 23353674 10.1038/nnano.2013.4

[b14] Archard J. F. Contact and rubbing of flat surfaces. J. Appl. Phys. 24, 981 (1953).

[b15] Greenwood J. A. & Tabor D. Deformation properties of friction junctions. Phys. Soc. Lond. B 68, 609–619 (1955).

[b16] Brockley C. & Fleming G. A model junction study of severe metallic wear. Wear 8, 374–380 (1965).

[b17] Chung K.-H. & Kim D.-E. Fundamental investigation of micro wear rate using an atomic force microscope. Tribol. Lett. 15, 135–144 (2003).

[b18] Liu J., Notbohm J. K., Carpick R. W. & Turner K. T. Method for characterizing nanoscale wear of atomic force microscope tips. ACS Nano 4, 3763–3772 (2010). 20575565 10.1021/nn100246g

[b19] Molinari J.-F., Ortiz M., Radovitzky R. & Repetto E. Finite element modeling of dry sliding wear in metals. Eng. Comput. 18, 592–610 (2001).

[b20] Spijker P., Anciaux G. & Molinari J.-F. Dry sliding contact between rough surfaces at the atomistic scale. Tribol. Lett. 44, 279–285 (2011).

[b21] Sha Z.-D. . Large-scale molecular dynamics simulations of wear in diamond-like carbon at the nanoscale. Appl. Phys. Lett. 103, 073118 (2013).

[b22] Pastewka L., Moser S., Gumbsch P. & Moseler M. Anisotropic mechanical amorphiza-tion drives wear in diamond. Nat. Mater. 10, 34–38 (2011). 21113152 10.1038/nmat2902

[b23] Bouchet M. D. B. . Energy filtering transmission electron microscopy and atomistic simulations of tribo-induced hybridization change of nanocrystalline diamond coating. Carbon 87, 317–329 (2015).

[b24] Bowden F. & Tabor D. The Friction and Lubrication of Solids Oxford University Press (2001).

[b25] McFarlane J. S. & Tabor D. Adhesion of solids and the effect of surface films. Proc. R. Soc. Lond. A 202, 224–243 (1950).

[b26] Celis B. D. Theoretical analysis of dry friction in brittle and ductile materials. Wear 116, 287–298 (1986).

[b27] Rabinowicz E. Influence of surface energy on friction and wear phenomena. J. Appl. Phys. 32, 1440–1444 (1961).

[b28] Rabinowicz E. The effect of size on the looseness of wear fragments. Wear 2, 4–8 (1958).

[b29] Rabinowicz E. Practical uses of the surface energy criterion. Wear 7, 9–22 (1964).

[b30] Rice J. R. Dislocation nucleation from a crack tip: an analysis based on the Peierls concept. J. Mech. Phys. Solids 40, 239–271 (1992).

[b31] Morse P. M. Diatomic molecules according to the wave mechanics. II. Vibrational levels. Phys. Rev. 34, 57–64 (1929).

[b32] Li A. & Szlufarska I. How grain size controls friction and wear in nanocrystalline metals. Phys. Rev. B 92, 075418 (2015).

[b33] Gnecco E., Bennewitz R. & Meyer E. Abrasive wear on the atomic scale. Phys. Rev. Lett. 88, 215501 (2002). 12059484 10.1103/PhysRevLett.88.215501

[b34] Sundaram N. K., Guo Y. & Chandrasekar S. Mesoscale folding, instability, and disruption of laminar flow in metal surfaces. Phys. Rev. Lett. 109, 106001 (2012). 23005303 10.1103/PhysRevLett.109.106001

[b35] Rabinowicz E. Friction and Wear of Materials Wiley pp 125–166 (1995).

[b36] Muser M. H. & Robbins M. O. in *Springer Handbook of Nanotechnology* (ed. Bhushan, B.) 717–738 (Springer, 2004).

[b37] Mate C. M. in *Tribology on the Small Scale: A Bottom Up Approach to Friction, Lubrication, and Wear* (ed. Mate, C. M.) 313–331 (Oxford Scholarship Online, 2007).

[b38] Maw W., Stevens F., Langford S. C. & Dickinson J. T. Single asperity tribochemical wear of silicon nitride studied by atomic force microscopy. J. Appl. Phys 92, 5103–5109 (2002).

[b39] Reches Z. & Lockner D. A. Fault weakening and earthquake instability by powder lubrication. Nature 467, 452–455 (2010). 20865001 10.1038/nature09348

[b40] Han R., Hirose T., Shimamoto T., Lee Y. & Ando J.-I. Granular nanoparticles lubricate faults during seismic slip. Geol. Soc. Am. Bull. 39, 599–602 (2011).

[b41] Hayashi N. & Tsutsumi A. Deformation textures and mechanical behavior of a hydrated amorphous silica formed along an experimentally produced fault in chert. GeoPhys. Res. Lett. 37, L12305 (2010).

[b42] Xu J. & Kato K. Formation of tribochemical layer of ceramics sliding in water and its role for low friction. Wear 245, 61–75 (2000).

[b43] Byerlee J. D. Theory of friction based on brittle fracture. J. Appl. Phys. 38, 2928–2934 (1967).

[b44] Wang D. F. & Kato K. Nano-scale fatigue wear of carbon nitride coatings: part I wear properties. J. Tribol. 125, 430–436 (2003).

[b45] Khonsari M. M. & Amiri M. Introduction to Thermodynamics of Mechanical Fatigue CRC press, 2012.

[b46] Mesarovic S. D. & Johnson K. Adhesive contact of elasticplastic spheres. J. Mech. Phys. Solids 48, 2009–2033 (2000).

[b47] Fischer-Cripps A. C. Introduction to Contact Mechanics 137–150Springer (2007).

[b48] Gao Y., Ruestes C. J., Tramontina D. R. & Urbassek H. M. Comparative simulation study of the structure of the plastic zone produced by nanoindentation. J. Mech. Phys. Solids 75, 58–75 (2015).

[b49] Griffith A. A. The phenomena of rupture and flow in solids. Philos. Trans. R. Soc. Lond. A 221, 163–198 (1921).

[b50] Tao Z. & Bhushan B. Surface modification of AFM silicon probes for adhesion and wear reduction. Tribol. Lett. 21, 1–16 (2006).

[b51] Chung K.-H. Wear characteristics of atomic force microscopy tips: a reivew. Int. J. Precision Eng. Manuf. 15, 2219–2230 (2014).

[b52] Bhushan B. & Sundararajan S. Micro/nanoscale friction and wear mechanisms of thin films using atomic force and friction force microscopy. Acta Mater. 46, 3793–3804 (1998).

[b53] Pei L., Hyun S., Molinari J. & Robbins M. O. Finite element modeling of elasto-plastic contact between rough surfaces. J. Mech. Phys. Solids 53, 2385–2409 (2005).

[b54] Luan B. & Robbins M. O. The breakdown of continuum models for mechanical contacts. Nature 435, 929–932 (2005). 15959512 10.1038/nature03700

[b55] Oliver W. & Pharr G. An improved technique for determining hardness and elastic modulus using load and displacement sensing indentation experiments. J. Mater. Res. 7, 1564–1583 (1992).

[b56] Mo Y., Turner K. T. & Szlufarska I. Friction laws at the nanoscale. Nature 457, 1116–1119 (2009). 19242472 10.1038/nature07748

[b57] Ziegenhain G., Urbassek H. M. & Hartmaier A. Influence of crystal anisotropy on elastic deformation and onset of plasticity in nanoindentation: a simulational study. J. Appl. Phys. 107, 061807 (2010).

[b58] Ashby M. & Jones D. Engineering Materials Pergamon (1980).

[b59] Pharr G., Oliver W. & Brotzen F. On the generality of the relationship among contact stiffness, contact area, and elastic modulus during indentation. J. Mater. Res. 7, 613–617 (1992).

[b60] Plimpton S. Fast parallel algorithms for short-range molecular dynamics. J. Comput. Phys 117, 1–19 (1995).

[b61] Stukowski A. Visualization and analysis of atomistic simulation data with OVITO-the open visualization tool. Model. Simul. Mater. Sci. Eng. 18, 015012 (2010).

[b62] Mendelev M., Kramer M., Becker C. & Asta M. Analysis of semi-empirical interatomic potentials appropriate for simulation of crystalline and liquid Al and Cu. Philos. Mag. 88, 1723–1750 (2008).

[b63] Mendelev M. I. . Devel-opment of new interatomic potentials appropriate for crystalline and liquid iron. Philos. Mag 83, 3977–3994 (2003).

[b64] Stillinger F. H. & Weber T. A. Computer simulation of local order in condensed phases of silicon. Phys. Rev. B 31, 5262–5271 (1985). 10.1103/physrevb.31.5262 9936488

[b65] Tersoff J. Modeling solid-state chemistry: Interatomic potentials for multicomponent systems. Phys. Rev. B 39, 5566–5568 (1989). 10.1103/physrevb.39.5566 9948964

[b66] Leung K., Pan Z. & Warner D. Atomistic-based predictions of crack tip behavior in silicon carbide across a range of temperatures and strain rates. Acta Mater 77, 324–334 (2014).

[b67] Pastewka L., Pou P., Perez R., Gumbsch P. & Moseler M. Describing bond-breaking processes by reactive potentials: Importance of an environment-dependent interaction range. Phys. Rev. B 78, 161402 (2008).

[b68] Vashishta P., Kalia R. K., Nakano A. & Rino J. P. Interaction potential for silicon carbide: a molecular dynamics study of elastic constants and vibrational density of states for crystalline and amorphous silicon carbide. J. Appl. Phys 101, 103515 (2007).

[b69] Archard J. F. & Hirst W. The wear of metals under unlubricated conditions. Phys. R. Soc. Lond. A 236, 397–410 (1956).

