# Supplementary material for: Critical length scale controls adhesive wear mechanisms
Source: Nat Commun. 2016 Jun 6;7:11816. doi: 10.1038/ncomms11816 (PMC4897754; doi:10.1038/ncomms11816)
Supplement: Supplementary Information — Supplementary Figures 1 - 11, Supplementary Tables 1 - 5 and Supplementary References [file ncomms11816-s1.pdf]

Supplementary Information

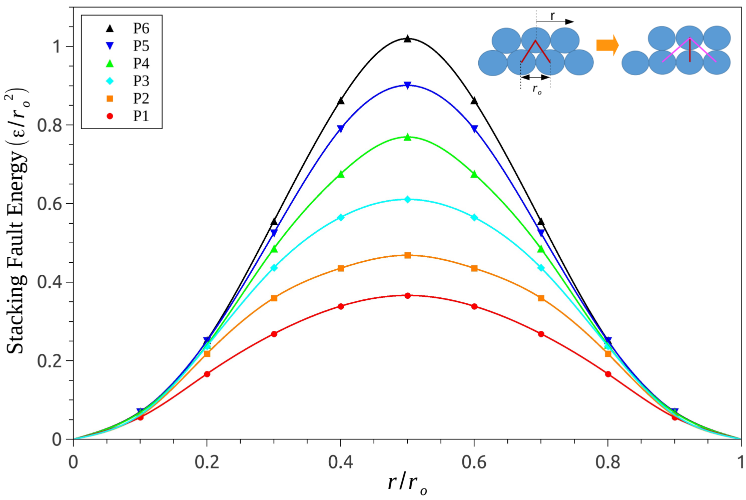

Supplementary Figure 1: **Unstable stacking fault energy of the model potentials.** Potential energy curves as a function of parallel stacking displacement for different potentials (P1-P6 in figure 2). The maximum value of energy represents the unstable stacking fault energy for each potential.

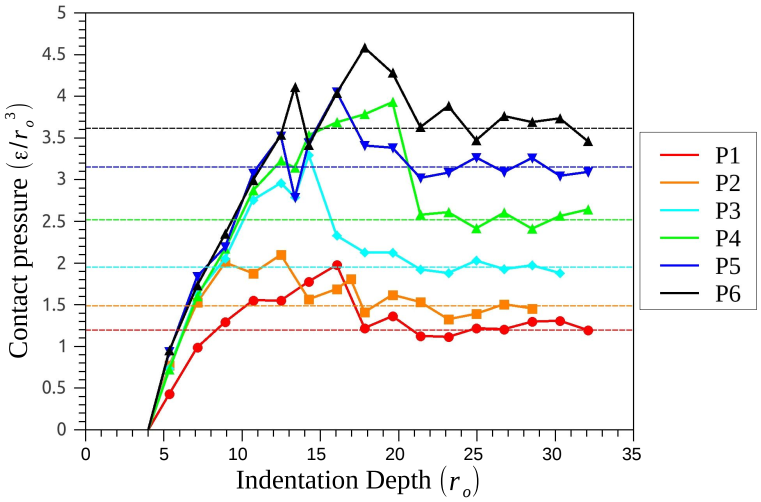

Supplementary Figure 2: **Hardness variation of the model potentials.** Contact pressure versus indentation depth obtained from the MD indentation simulations. The contact pressure is computed as the indentation force (see the inset of figure 2) divided by the projected contact area. The peak in each curve corresponds to the first dislocation nucleation. It is followed by rather constant contact pressure due to continued nucleation of dislocations. The constant value is considered to be the hardness. see Supplementary Table 1

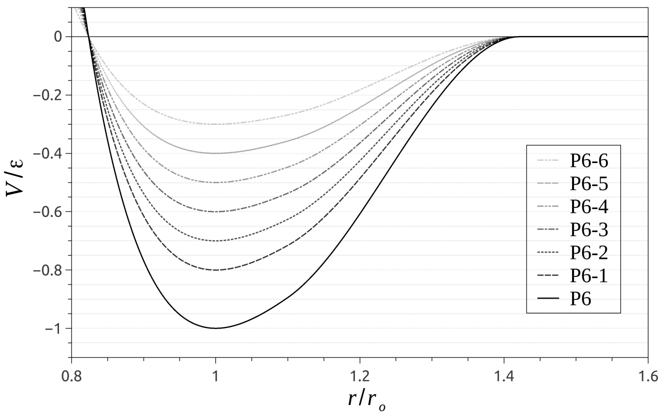

Supplementary Figure 3: **Interfacial model potentials.** Interfacial potential energy curves corresponding to the brittle potential (P6), which controls the adhesion between the sliding surfaces. See Supplementary Table 2 for further information.

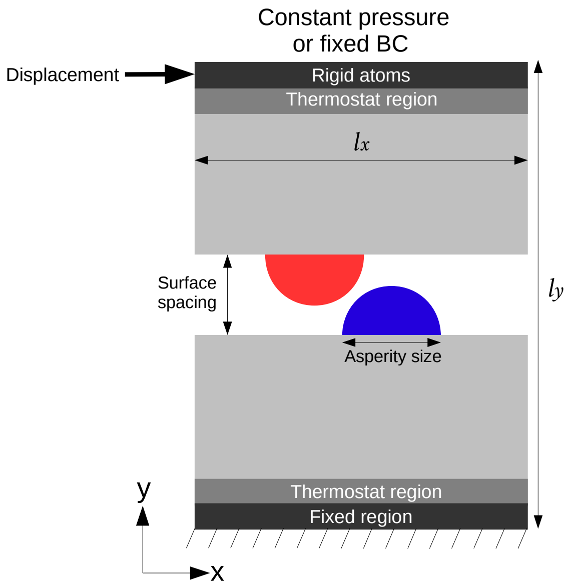

Supplementary Figure 4: **Simulation geometry and boundary conditions.** Two extreme boundary conditions were applied on the top boundary, vertically fixed and constant applied pressure.

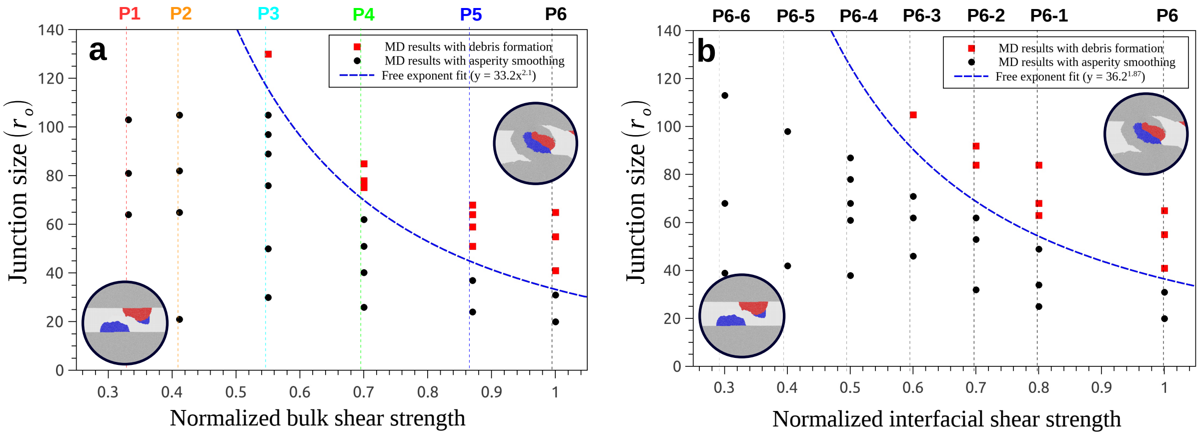

(a) **(b)**

Supplementary Figure 5: **Individual contribution of bulk and interfacial shear strength.** In (**a**), the data corresponds to cases of full interfacial adhesion, whereby the junction strength is governed by the bulk shear strength. This figure clearly shows that a more ductile potential needs a larger junction size to produce a fracture-induced debris particle. In (**b**), the data corresponds to the cases of reduced interfacial adhesion, whereby the junction strength is governed by the shear strength of the interfacial adhesion. In (**a**), the x-axis is normalized by the max bulk shear strength whereby in (**b**) it is normalized by the bulk shear strength for the particular case (see Supplementary Tables 1 and 2). Vertical dash lines show the corresponding potential for each simulation point where colors are consistent with figure 2. This figure demonstrates that the critical junction size scales with σ_j^{-2}, consistent with equation 3, and independent of whether σ_j is governed by the shear strength of the bulk material or the interfacial shear strength.

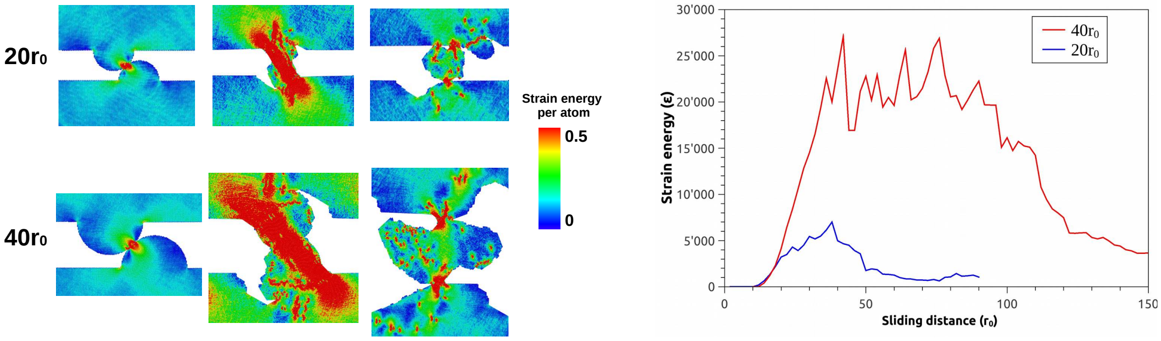

(a) (b)

Supplementary Figure 6: **Scalability of the released elastic energy.** (**a**) shows the debris formation in two simulations with different initial asperity sizes ($20r_0$ and $40r_0$) and the strain energy evolution per atom. (**b**) compares the corresponding evolution of total strain energy during the debris formation event. As shown, a simulation with twice as large junction size displayed approximately four times greater release in strain energy upon debris particle formation, which confirms the scalability of stored strain energy with the junction size (see equation 1). Note that the scalability relation is $E_{el} \sim d^2$ in 2D and $E_{el} \sim d^3$ for 3D, which is consistent with previous literature.[1,2]

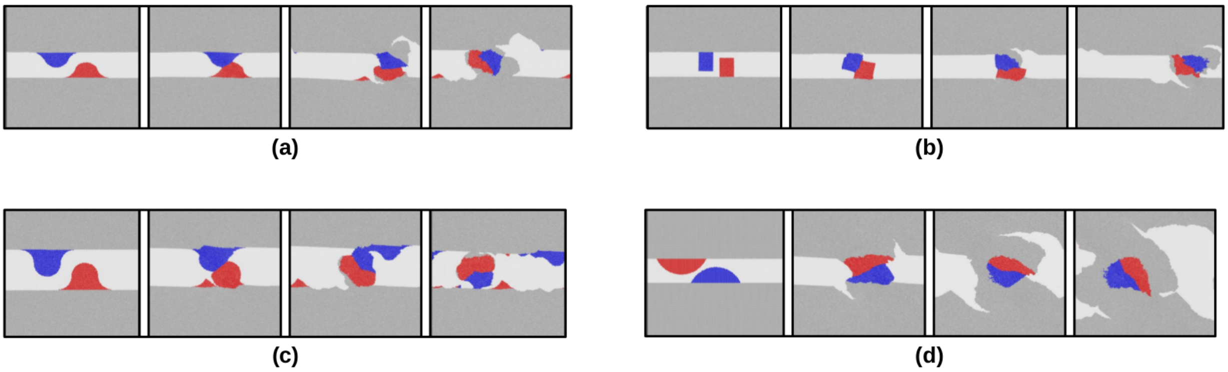

Supplementary Figure 7: **Influence of asperities shape and configuration.** Atomistic simulation results of dry sliding with the most brittle (P6) potential for four initial geometries. Severe shape changing plastic deformation occurs at the onset of contact in all cases. Thus, the initial geometry does not have a considerable effect on wear debris formation mechanism at the asperity level. Note that we compute the junction size as the true contact area between asperities when the contact angle and the tangential forces reach zero and maximum values, respectively.

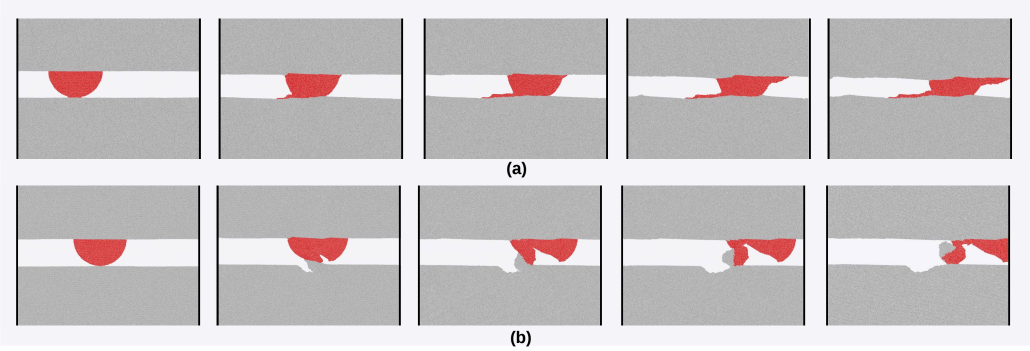

Supplementary Figure 8: **Adhesive wear mechanisms transition during sliding between a single asperity and a flat surface.** Atomistic simulation results of dry sliding between a single asperity and a flat surface with (**a**) the most ductile (P1) and (**b**) the most brittle potential (P6). The transition in adhesive wear mechanisms is observed similar to the interlocking asperities cases (figure 3). Figure b clearly shows the correlation of the worn volume with the asperity junction size (and not the asperity size), consistent with the analytic model. This outcome supports the geometrical and configuration independency of the adhesive wear mechanisms.

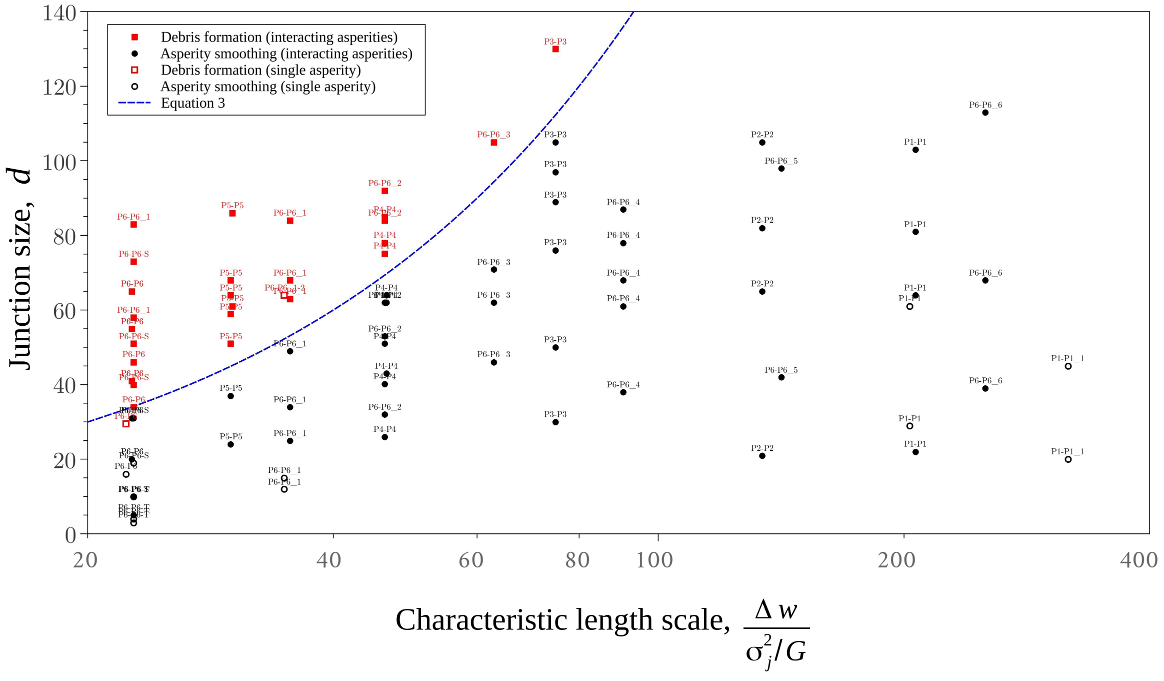

Supplementary Figure 9: **Detailed version of figure 4**. First label represents the body potential while the second shows the interfacial potential. The third label (if any) displays asperity shape where T or S stands for triangular and square initial asperity shapes. Empty symbols represent single asperity simulations, while full symbols display simulations with interlocking asperities. The logarithmic x axis is used for a better representation of simulations' labels.

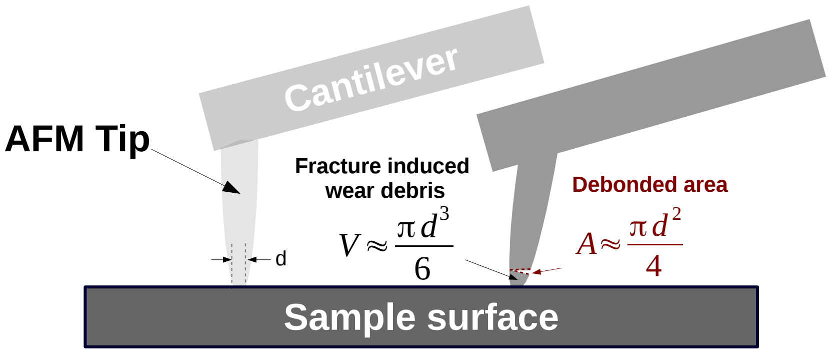

Supplementary Figure 10: **Schematic of fracture-induced debris formation in an adhesive AFM wear experiment.** Having equations 1-3 and assuming $\alpha = \beta = 1$ (which is corresponding to the removal of an idealized 3D spherical particle from the AFM tip), $\lambda = 3$ is obtained for a 3D case. This value is used to estimate the critical junction size for different AFM tip materials in table 4.

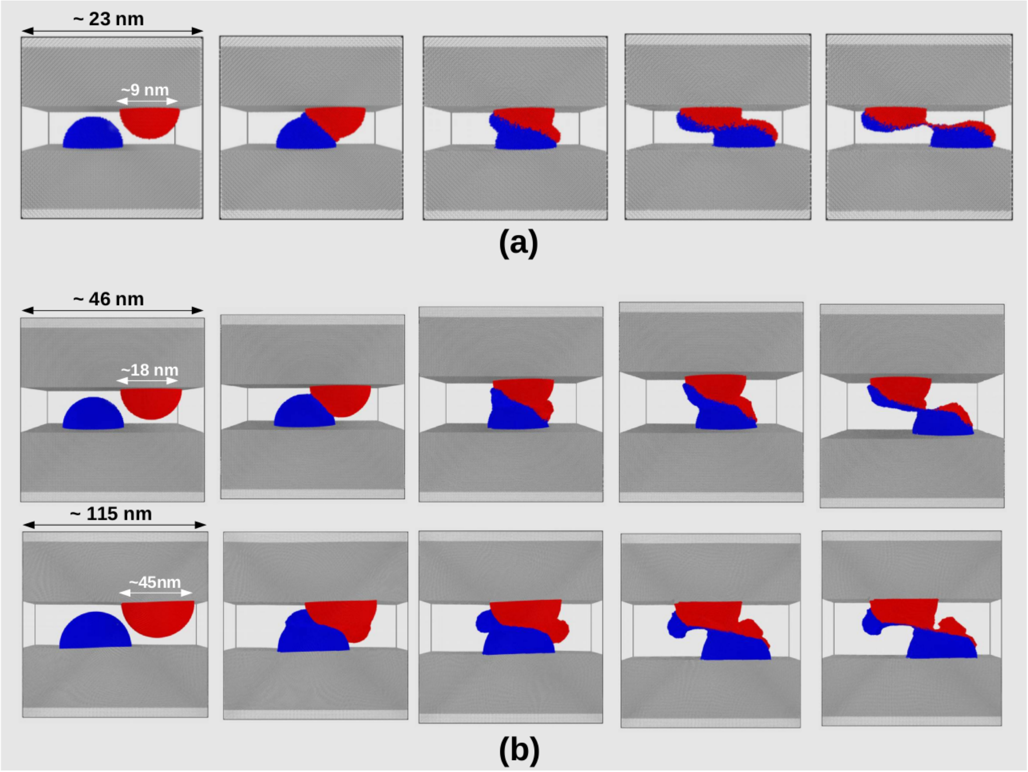

Supplementary Figure 11: **Absence of fracture-induced debris formation in 3D simulations with standard potentials due to the length scale constraint.** Snapshots from largescale 3D simulation with (**a**) Si Tersoff potential[3] and (**b**) Fe EAM potential[4] showing lack of debris formation in first asperity collision. The same interatomic potentials are used to model the interaction between surface atoms (i.e. full adhesion). The coloring of atoms is artificial and for better visualization of the wear mechanisms.

Supplementary Table 1: Parameters of body potentials.

Potential label	Normalized cut off radius, r_{cut}/r_o	Surface energy, γ_{surf}	Unstable stacking fault energy, γ_{usf}	$\frac{\gamma_{\mathrm{surf}}}{\gamma_{\mathrm{usf}}}$	Shear modulus, G	Shear Strength, τ	Normalized Shear Strength τ/τ_{P6}
-	-	$(\varepsilon\, r_o^{-1})$	$(\varepsilon\, r_o^{-1})$	-	$(\varepsilon\, r_o^{-3})$	$(\varepsilon\, r_o^{-3})$	-
P1	1.71	1.0	0.37	2.70	2.7	0.23	0.33
P2	1.60	1.0	0.47	2.12	2.7	0.29	0.41
P3	1.56	1.0	0.61	1.64	2.7	0.39	0.55
P4	1.47	1.0	0.77	1.43	2.7	0.49	0.70
P5	1.44	1.0	0.90	1.11	2.7	0.60	0.87
P6	1.42	1.0	1.0	1.0	2.7	0.69	1.0

Supplementary Table 2: Parameters of interfacial potentials.

Potential label<br>-	Normalized cut off radius, r_{cut}/r_o<br>-	Surface energy, γ_{surf}<br>$(\varepsilon\, r_o^{-1})$	Unstable stacking fault energy, γ_{usf}<br>$(\varepsilon\, r_o^{-1})$	$\frac{\gamma_{\mathrm{surf}}}{\gamma_{\mathrm{usf}}}$<br>-
P6-1	1.42	0.8	0.8	1.0
P6-2	1.42	0.7	0.7	1.0
P6-3	1.42	0.6	0.6	1.0
P6-4	1.42	0.5	0.5	1.0
P6-5	1.42	0.4	0.4	1.0
P6-6	1.42	0.3	0.3	1.0

Supplementary Table 3: Parameters examined by atomistic simulations. All units are given in reduced Lennard-Jones units.

Parameter	Value
l_x	200-600
l_y	400-1000
asperity size	20-140
spacing	10-100
load per atom	0.00-0.01
velocity	0.001-0.05

Supplementary Table 4: Physical properties of common AFM probe materials and the corresponding critical junction size estimated by equation 3. Bold numbers in the last column are the average of the values found in literature. σ_j is estimated from hardness using the relation on page S3. For Diamond, the critical shear strength is equal to hardness.[5,7]

AFM tip Material	Elastic modulus (GPa)	Poisson's Ratio -	Nanohardness (GPa)	Surface energy ($N\,m^{-1}$)	Estimated critical junction size from equation 3
Diamond	930-1200[5,6]	0.007-0.115[5]	60-90[5–7]	4.1-6.2[8,9]	2-6.5nm (**4**nm)
Silicon nitride	230-280[10–12]	0.20-0.27[13,14]	20-37[10,11]	1.1[15]	10-40nm (**22**nm)
Silicon	107-130[16,17]	0.27-0.35[18]	13[19]	1.5-2[8,20]	56-98 nm (**76**nm)
Gold	80[21]	0.40	1-2[21]	1-1.2[22]	1-5 mm (**3**mm)
Silver	83[23]	0.38	1-1.7[23]	1.1[24]	2-6 mm (**4**mm)

Supplementary Table 5: Detailed information of AFM wear experiments available in literature and corresponding wear mechanisms.

Authors	AFM tip material	Substrate material	Applied load (nN)	AFM tip Diameter (nm)	Observed wear mechanism	Remarks
Liu et al.,[25]	Si	UNCD	Only adhesive force	40 ± 10 (TEM)	Continuous gradual wear	High work of adhesion
				50 ± 10 (TEM)	Tip fracture	Room temperature $\sim$ 20-50% RH
	SiN_x	UNCD	Only adhesive force	50 ± 10 (TEM)	Tip fracture	
				90 ± 20 (TEM)	Tip fracture	
Chung and Kim,[19]	Si	Si	10.8	40 ± 10 (SEM)	Continuous gradual wear	Room temperature $\sim$ 20% RH
	Si	Si	104.5-325	570 ± 200 (WV)	Tip fracture	
	Si_3N_4	Si	21	40 ± 10 (SEM)	Tip fracture	
Chung et al.,[26]	Si	Si	10	20 ± 10 (TEM)	Gradual wear	Room temperature
Gotsmann and Lantz[27]	Si	Polymer	5-100	40 ± 20 (TEM)	Gradual wear (No sign of fracture)	Room temperature
Vahdat et al.,[12]	SiN_x	UNCD	30-60	27 (TEM)	Gradual wear by plasticity	Room temperature$\sim$ 15% RH
Chung[28]	Si	Si	100	10 (TEM)	Gradual wear	Room temperature
	SiN_x	Si	10	6 (TEM)	Gradual wear	Room temperature
Jacobs and Carpick[29]	Si	Diamond	Only adhesive force	15 ± 5 (TEM)	Gradual wear	vacuum
Tao and Bhushan[30]	Si	Si	100-300	40 (TEM)	Gradual wear	vacuum
Khurshudov and Kato[31]	Si_3N_4	Si	10	20-40	Tip fracture	Air
Sato et al.,[32]	Ag	Ag	Only adhesive force	2-12 (TEM)	Gradual wear	Ultra-high vacuum
Merkle et al.,[33]	Au	Au	Probably only adhesive force (normal force could not be directly measured)	4-200 (TEM)	liquid-like behavior (plastic flow)	Ultra-high vacuum

References

(1) Mesarovic, S. D.; Johnson, K. Adhesive contact of elasticplastic spheres. *J. Mech. Phys. Solids* **2000**, *48*, 2009 – 2033.

(2) Fischer-Cripps, A. C. *Introduction to Contact Mechanics*; Springer, 2007; Chapter Elastic-Plastic Indentation Stress Fields, pp 137–150.

(3) Tersoff, J. Modeling solid-state chemistry: Interatomic potentials for multicomponent systems. *Phys. Rev. B* **1989**, *39*, 5566–5568.

(4) Mendelev, M. I.; Han, S.; Srolovitz, D. J.; Ackland, G. J.; Sun, D. Y.; Asta, M. Development of new interatomic potentials appropriate for crystalline and liquid iron. *Philos. Mag.* **2003**, *83*, 3977–3994.

(5) Hess, P. The mechanical properties of various chemical vapor deposition diamond structures compared to the ideal single crystal. *J. Appl. Phys.* **2012**, *111*.

(6) Telling, R. H.; Pickard, C. J.; Payne, M. C.; Field, J. E. Theoretical Strength and Cleavage of Diamond. *Phys. Rev. Lett.* **2000**, *84*, 5160–5163.

(7) Zhang, T.; Huan, Y. Nanoindentation and nanoscratch behaviors of {DLC} coatings on different steel substrates. *Compos. Sci. Technol.* **2005**, *65*, 1409 – 1413.

(8) Zhang, J.-M.; Ma, F.; Xu, K.-W.; Xin, X.-T. Anisotropy analysis of the surface energy of diamond cubic crystals. *Surf. Interface Anal.* **2003**, 805–809.

(9) Hong, S.; Chou, M. Y. Effect of hydrogen on the surface-energy anisotropy of diamond and silicon. *Phys. Rev. B* **1998**, *57*, 6262–6265.

(10) Zerr, A.; Kempf, M.; Schwarz, M.; Kroke, E.; Goken, M.; Riedel, R. Elastic Moduli and Hardness of Cubic Silicon Nitride. *J. Am. Ceram. Soc.* **2002**, *85*, 86–90.

(11) Chung, K.-H.; Kim, D.-E. Wear characteristics of diamond-coated atomic force microscope probe. *Ultramicroscopy* **2007**, *108*, 1–10.

(12) Vahdat, V.; Grierson, D. S.; Turner, K. T.; Carpick, R. W. Mechanics of Interaction and Atomic-Scale Wear of Amplitude Modulation Atomic Force Microscopy Probes. *ACS Nano* **2013**, *7*, 3221–3235.

(13) Khan, A.; Philip, J.; Hess, P. Youngs modulus of silicon nitride used in scanning force microscope cantilevers. *J. Appl. Phys.* **2004**, *95*, 1667–1672.

(14) Lynch, C. T. *Materials Science and Engineering Handbook*; CRC Press, 1989; p 321.

(15) Stephan, A.; Finot, E.; Ji, H.-F.; Pinnaduwage, L.; Thundat, T. Micromechanical measurement of active sites on silicon nitride using surface free energy variation. *Ultramicroscopy* **2002**, *91*, 1–8, Proceedings of the third International Conference on Scaning Probe Microscopy, Sensors and Nanostructures.

(16) Khurshudov, A. G.; Kato, K.; Koide, H. Wear of the AFM diamond tip sliding against silicon. *Wear* **1997**, *203204*, 22–27, 11th International Conference on Wear of Materials.

(17) Wortman, J. J.; Evans, R. A. Young's Modulus, Shear Modulus, and Poisson's Ratio in Silicon and Germanium. *J. Appl. Phys.* **1965**, *36*, 153–156.

(18) Hess, P. Laser diagnostics of mechanical and elastic properties of silicon and carbon films. *Appl. Surf. Sci.* **1996**, *106*, 429–437, Proceedings of the Second International Conference on Photo-Excited Processes and Applications.

(19) Chung, K.-H.; Kim, D.-E. Fundamental Investigation of Micro Wear Rate Using an Atomic Force Microscope. *Tribol. Lett.* **2003**, *15*, 135–144.

(20) Jaccodine, R. J. Surface Energy of Germanium and Silicon. *J. Electrochem. Soc.* **1963**, *110*, 524–527.

(21) Dietiker, M.; Nyilas, R. D.; Solenthaler, C.; Spolenak, R. Nanoindentation of single-crystalline gold thin films: Correlating hardness and the onset of plasticity. *Acta Mater.* **2008**, *56*, 3887–3899.

(22) Needs, R. J.; Mansfield, M. Calculations of the surface stress tensor and surface energy of the (111) surfaces of iridium, platinum and gold. *J. Phys.-Condens Mater.* **1989**, *1*, 7555–7563.

(23) Almasri, A. H.; Voyiadjis, G. Nano-indentation in FCC metals: experimental study. *Acta Mech..* **2010**, *209*, 1–9.

(24) Sundquist, B. A direct determination of the anisotropy of the surface free energy of solid gold, silver, copper, nickel, and alpha and gamma iron. *Acta Metal.* **1964**, *12*, 67–86.

(25) Liu, J.; Notbohm, J. K.; Carpick, R. W.; Turner, K. T. Method for Characterizing Nanoscale Wear of Atomic Force Microscope Tips. *ACS Nano* **2010**, *4*, 3763–3772.

(26) Chung, K.-H.; Lee, Y.-H.; Kim, D.-E. Characteristics of fracture during the approach process and wear mechanism of a silicon AFM tip. *Ultramicroscopy* **2005**, *102*, 161–171.

(27) Gotsmann, B.; Lantz, M. A. Atomistic Wear in a Single Asperity Sliding Contact. *Phys. Rev. Lett.* **2008**, *101*, 125501.

(28) Chung, K.-H. Wear characteristics of atomic force microscopy tips: A reivew. *Int. J. Precis. Eng. Man.* **2014**, *15*, 2219–2230.

(29) Jacobs, T. D. B.; Carpick, R. W. Nanoscale wear as a stress-assisted chemical reaction. *Nat. Nanotechnol.* **2013**, *8*, 108–112.

(30) Tao, Z.; Bhushan, B. Surface modification of AFM silicon probes for adhesion and wear reduction. *Tribol. Lett.* **2006**, *21*, 1–16.

(31) Khurshudov, A.; Kato, K. Wear of the atomic force microscope tip under light load, studied by atomic force microscopy. *Ultramicroscopy* **1995**, *60*, 11–16.

(32) Sato, T.; Ishida, T.; Jalabert, L.; Fujita, H. Real-time transmission electron microscope observation of nanofriction at a single Ag asperity. *Nanotechnology* **2012**, *23*, 505701.

(33) Merkle, A. P.; Marks, L. D. Liquid-like tribology of gold studied by in situ TEM. *Wear* **2008**, *265*, 1864–1869.
